# Modulation of T-type Ca^2+^ channels by Lavender and Rosemary extracts

**DOI:** 10.1371/journal.pone.0186864

**Published:** 2017-10-26

**Authors:** Chaymae El Alaoui, Jean Chemin, Taoufiq Fechtali, Philippe Lory

**Affiliations:** 1 Institut de Génomique Fonctionnelle, CNRS, INSERM, University of Montpellier, Montpellier, France; 2 LabEx 'Ion Channel Science and Therapeutics', Montpellier, France; 3 Laboratoire de Neurosciences, Physiopathologies Intégrées et Substances Naturelles, Faculté des Sciences et Techniques Mohammedia, Université Hassan II, Casablanca, Morocco; University of Waterloo, CANADA

## Abstract

Medicinal plants represent a significant reservoir of unexplored substances for early-stage drug discovery. Of interest, two flowering Mediterranean plants have been used for thousands of years for their beneficial effects on nervous disorders, including anxiety and mood. However, the therapeutic potential of these plants regarding their ability to target ion channels and neuronal excitability remains largely unknown. Towards this goal, we have investigated the ability of Lavender and Rosemary to modulate T-type calcium channels (TTCCs). TTCCs play important roles in neuronal excitability, neuroprotection, sensory processes and sleep. These channels are also involved in epilepsy and pain. Using the whole-cell patch-clamp technique, we have characterized how Lavender and Rosemary extracts, as well as their major active compounds Linalool and Rosmarinic acid, modulate the electrophysiological properties of recombinant TTCCs (Ca_V_3.2) expressed in HEK-293T cells. Both the methanolic and essential oil extracts as well as the active compounds of these plants inhibit Ca_v_3.2 current in a concentration-dependent manner. In addition, these products also induce a negative shift of the steady-state inactivation of Ca_V_3.2 current with no change in the activation properties. Taken together, our findings reveal that TTCCs are a molecular target of the Lavender and Rosemary compounds, suggesting that inhibition of TTCCs could contribute to the anxiolytic and the neuroprotective effects of these plants.

## Introduction

Medicinal plants have been identified and used throughout human history [1]. Because of their ability to synthesize a wide variety of chemical compounds (alkaloids, polyphenolics, terpenoids, fatty acids and lipids, etc.) either for their normal development or against stressful and threatening conditions, they have been suggested to be an interesting pharmaceutical industry. Moreover, because of the potential side and adverse effects of synthetic drugs, scientists interested in drug discovery have turned their attention to herbal medicines as effective lead compounds for the management of health ailments including inflammatory, cardiovascular and neurological disorders[2, 3]. It’s worth noting that 49% of the new chemical drugs that were introduced between 1991 and 2002 had a natural origin witnessing the popularity of medicinal plants use worldwide [4].

Lavender and Rosemary are the most popular medicinal plants cultivated and grown nowadays. Native to the Mediterranean basin and Southern Europe, they have been used either dried or as essential oil for a variety of culinary, cosmetic and therapeutic purposes [5–7]. Studies have reported the existence of approximately 28 species and over than 200 varieties of Lavender (*Lavandula sp*.). The genus Lavender belongs to the Labiatae/Lamiaceae family and is divided into four main species: *Lavandula latifolia*, *Lavandula angustifolia* (*LA*)*; Lavandula stoechas* (*LS*) *and Lavandula x intermedia* [8]. Lavender essential oil is generally produced by steam distillation and contains a complex mixture of mono- and sesquiterpenoid alcohols, esters, oxides, and ketones, in which the major components are the monoterpenoids linalool and linalyl acetate [5]. Lavender oil was suggested to possess anticonvulsant, anxiolytic, analgesic and neuroprotective properties [5, 9–11].

Rosemary (*Rosmarinus officinalis*, *RO*) is one of the most interesting medicinal plants known for its promising medicinal use. *Rosmarinus officinalis* (Lamiaceae) oil consists of high percentages of biologically active compounds such as phenolic acids (Rosmarinic acid, chlorogenic acid), phenolic diterpenes (e.g. carnosic acid, carnosol), and flavonoids (e.g. derivatives of apigenin and luteolin) [12, 13]. A developing body of evidence suggests Rosemary to be a powerful remedy for various medical purposes thanks to its anti-oxidant, antinociceptive, and neuroprotective properties [14–17]. Rosmarinic acid, one of the major components of *RO*, is a polyphenolic compound and has been shown to possess anti-inflammatory, anti-oxidant and anxiolytic/antidepressive-like properties [18–20].

The precise mode of action of these two medicinal plants remains unclear. Studies to unveil the molecular mechanisms implicated in their therapeutical effects have recently suggested the modulation of GABAergic [21], serotonergic neurotransmission [22], as well as voltage-gated calcium channels including high voltage-activated (HVA) calcium channels by *Lavandula angustifolia* [10]. However, it has not been investigated whether Lavender (*Lavandula angustifolia* and *Lavandula stoechas*) and Rosemary can also affect low voltage-activated (LVA), T-type calcium channels (TTCCs).

Compared to HVA calcium channels, TTCCs are specifically activated by small membrane depolarization that allow calcium entry near the cell membrane resting potential [23, 24]. Heterogeneity in the functional properties of TTCCs is supported by molecular studies that have described three genes encoding these channels: the Ca_V_3.1, Ca_V_3.2, and Ca_V_3.3 subunits [23, 25]. These subunits are differentially expressed throughout the body, especially in the brain [26]. TTCCs are broadly involved in many physiological processes including sleep [27], proliferation [28, 29], neuronal firing, epilepsy [30, 31] and pain [32, 33]. Furthermore, recent studies have reported TTCCs to be an interesting molecular target for various natural substances like bioactive lipids and lipoaminoacids [34–36], toxins [37] and natural products from plants including the genera Cannabis, Curcuma and Syzygium [38–40].

In the present study, we have searched for plant extracts modulating TTCCs and we describe the pharmacological inhibition of TTCCs by Lavender and Rosemary using Cav3.2 channels expressed in HEK-293T.

## Materials and methods

### Ethics statement

*Lavandula stoechas*, *Rosmarinus officinalis*, *Ricinus cummunis* and *Citrullus colocynthis* were collected at National Institute of Agronomic Research (INRA), Agadir, Morocco. No specific permissions were required for these locations/activities. The botanical identity of each plant was determined and authenticated by Dr. R. Bouharroud, taxonomist at INRA, Agadir, Morocco.

### Methanolic extraction protocol

Fresh plant materials were dried at 40°C during 48 to 96 hours, then homogenized to fine powder by grinding and sieving until the stabilization of weight. 20 g of dried plant materials were extracted with 200 ml of pure methanol and kept on a rotary shaker for maceration for a total duration of 72h. Thereafter, the extracts were filtered and evaporated to dryness in Rotavapor^®^ vacuum (60 rpm at 40°C). The final extracts were stored at 4°C for further studies.

### Plant essential oil and active principles

Essential oils of *Lavandula steachas*, *Lavandula angustifolia Miller* and *Rosmarinus officinalis* were purchased from Vitalba (Sartène, France). Rosmarinic acid (RA) and Linalool were purchased from Sigma-Aldrich.

### Cell culture and transfection protocols

HEK-293T cells stably expressing the Ca_V_3.2 channels isoform (kindly provided by Dr. E. Perez-Reyes, University of Virginia) were cultivated in Dulbecco’s Modified Eagle’s Medium supplemented with GlutaMax, 400μg/ml G418 (Life Technologies) and 10% fetal bovine serum (Invitrogen). In some experiments, HEK-293T cells transfection with plasmids expressing human Ca_V_3 constructs was performed using jet-PEI (QBiogen) with a DNA mix containing 0.5% of a GFP encoding plasmid and 99.5% of the Cav3 constructs. Two days after transfection, HEK-293T cells were dissociated with Versene (Invitrogen) and plated at a density of ~35x10^3^ cells in 35 mm Petri dish for electrophysiological recordings performed the following day.

### Electrophysiological recordings

Whole-cell calcium currents were recorded at room temperature using an Axopatch 200B amplifier (Molecular Devices). For recording macroscopic T-type calcium currents, the extracellular solution contained the following (in mM): 135 NaCl, 20 TEACl, 2 CaCl_2_, 1 MgCl_2_, and 10 HEPES (pH adjusted to 7.25 with KOH, ~330 mOsm). Borosilicate glass pipettes have a typical resistance of 1.5–2.5 MOhm when filled with the internal solution containing the following (in mM): 140 CsCl, 10 EGTA, 10 HEPES, 3 Mg-ATP, 0.6 GTPNa, and 3 CaCl_2_ (pH adjusted to 7.25 with KOH ~315 mOsm). Recordings were filtered at 2 kHz. During the Ca_V_3.2 current recordings, the chamber was constantly perfused (~100 μl/min) with the control or with the drug solutions using a gravity-driven homemade perfusion device. Data were analyzed using the pCLAMP9 (Molecular devices) and GraphPad Prism (GraphPad) softwares. The dose-response curves were obtained from fitting data to the Hill equation, I/IMAX = 100/(1+10^((LogIC50-Log[compound])*HillSlope). Current-voltage (*I-V)* curves were fitted using a combined Boltzmann and linear Ohmic relationships, where *I = G*_*max*_ *x (V*_*m*_*-V*_*rev*_*)/(1+exp((V*_*m*_*-Vm*_*0*.*5*_*)/slope factor))*. Correspondingly, steady-state inactivation curves were fitted using the Boltzmann equation where *I/Imax = 1/(1+exp((V*_*m*_*-Vm*_*0*.*5*_*)/slope factor)*.

### Statistical analysis

Results are presented as the mean ± SEM, and *n* is the number of cells used. Statistical significance was evaluated by Student’s unpaired t-test (* P<0.05, ** P<0.01 and *** P<0.001)

## Results

### Modulation of Ca_V_3.2 calcium channels by medicinal plant methanolic extracts

**Fig 1:**
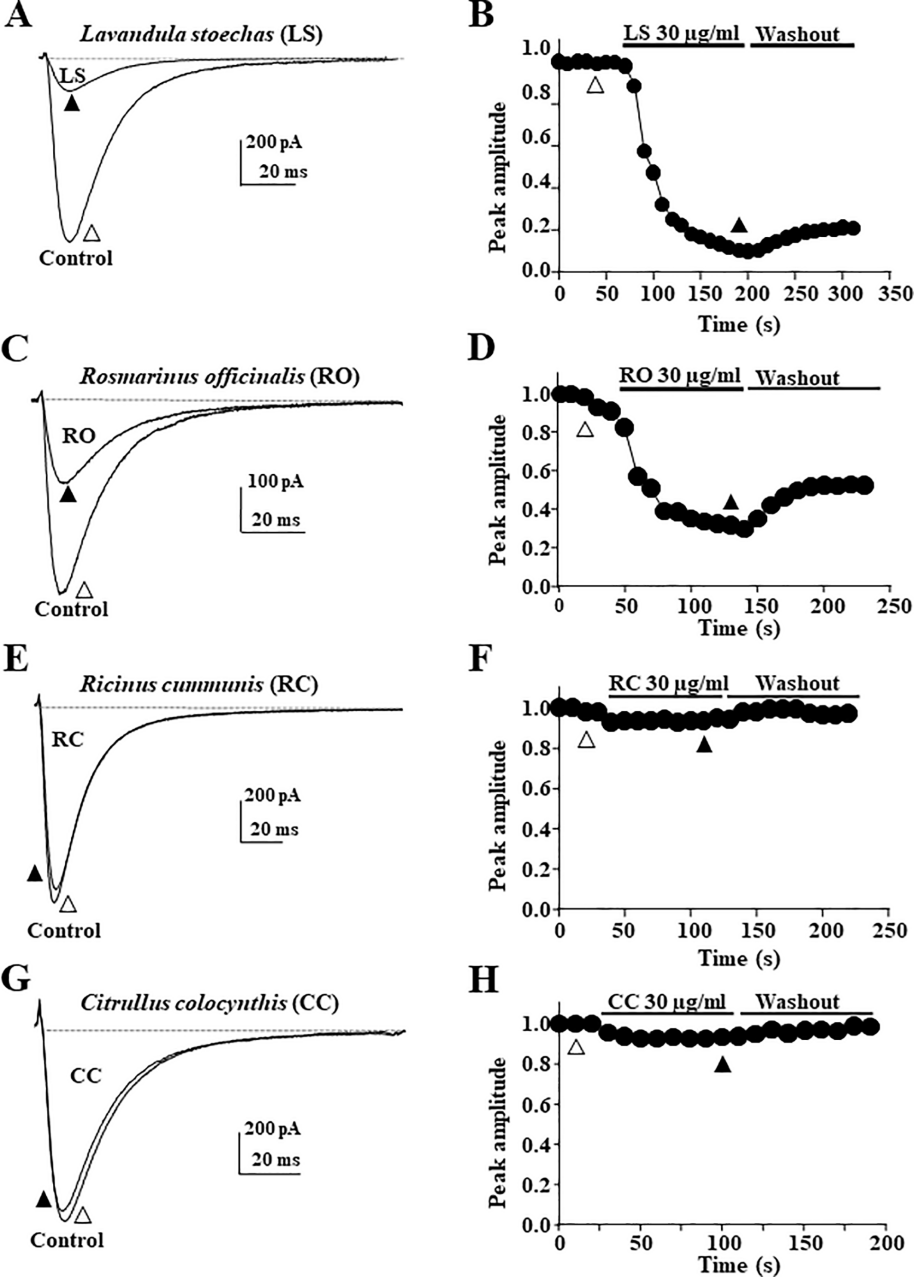
Modulation of Ca_V_3.2 channels by medicinal plant methanolic extracts. Whole-cell patch clamp recordings of T-type calcium current were obtained on HEK-293T cells stably expressing recombinant human Ca_V_3.2 channels. Currents were elicited by stepping from a holding potential (HP) of -80 mV to a test pulse (TP) of -30 mV applied every 10 seconds. Effect of the methanolic extracts (30 μg/ml) of the medicinal plants *Lavandula stoechas* (**A-B**), *Rosmarinus officinalis* (**C-D**), *Ricinus cummunis* (**E-F**), or *Citrullus colocynthis* (**G-H**) are illustrated with representative current traces collected before (open triangle) and during bath application (filled triangle) of the extracts (left panels). The corresponding time plots (right panels) illustrate the time-course of the inhibitory effect and washout of the extracts. Each extract panel is representative of 6 to 7 experiments.

In a first set of experiments, we tested the ability of several Mediterranean medicinal plants to modulate TTCCs. These experiments were performed using recombinant Ca_V_3.2 channels. Ca_V_3.2 channel modulation was determined by measuring the T-type current in whole cell configuration on cells stepped from -80 to -30 mV following superfusion of the extracts at a concentration of 30 μg/ml. Fig 1 illustrates the efficacy of four methanolic plant extracts by showing typical Ca_V_3.2 current trace recordings and the corresponding time plots. Application of *Lavandula stoechas* (*LS*) inhibited significantly Ca_V_3.2 channels (Fig 1A and 1B). The average current inhibition induced by 30 μg/ml of *LS* was 85% (I/Ictrl = 15 ± 5.2% *p<0*.*01*, n = 6). *LS* developed a fast inhibitory effect that did not readily reverse upon wash-out. Similarly, a 42% inhibition (I/Ictrl = 68 ± 2% *p<0*.*01*, n = 6) was obtained following application of the methanolic extract of *Rosmarinus officinalis* (*RO*) on Ca_V_3.2 currents (Fig 1C and 1D). On the contrary, no significant inhibition was obtained after the application of *Ricinus cummunis* (*RC*) (Fig 1E and 1F, I/Ictrl = 95.7 ± 1.5%, n = 7) and *Citrullus colocynthis* (*CC*) extracts (Fig 1G and 1H, I/Ictrl = 95.4 ± 2.1%, n = 6). These data led us to further investigate the efficacy of *Lavandula* and *Rosmarinus* species to modulate Ca_V_3.2 channels.

### Ca_V_3.2 channel inhibition by Lavandula species and Linalool is concentration-dependent

**Fig 2:**
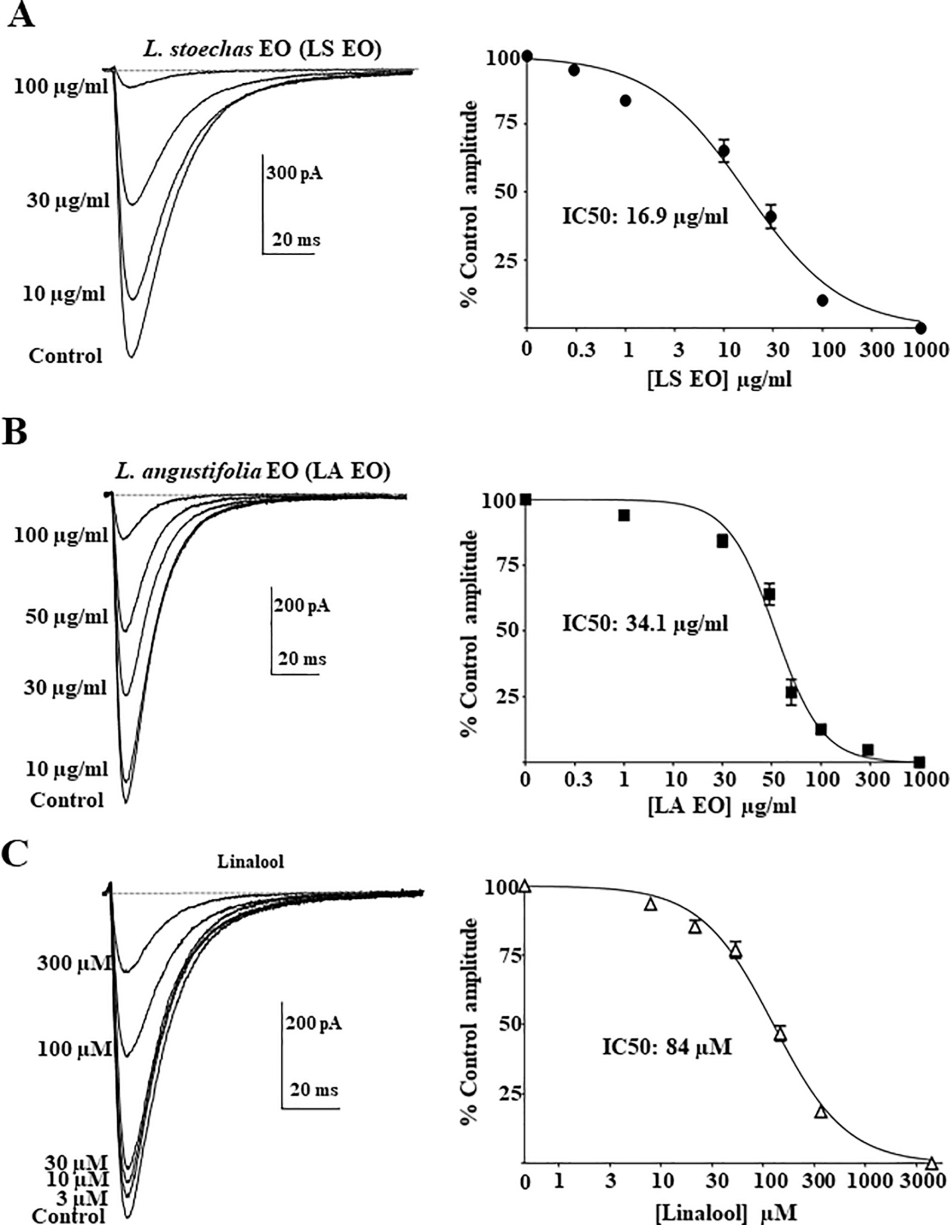
Inhibition of Ca_V_3.2 channel by *Lavandula* essential oils and Linalool. Dose-response curves of the inhibitory effect of *Lavandula steochas* (*LS*) (**A**) *Lavandula angustifolia Miller* (*LA*) *(officinalis)* (**B**) and Linalool (**C**) on Ca_V_3.2 current. Inhibition of Ca_V_3.2 channel currents was obtained by serial increase in concentrations of *Lavandula sp*. extracts. The IC_50_ for Linalool (84 μM) corresponds to ~12.6 μg/ml. Percentages of inhibition were averaged and plotted against compound concentrations (right panels; n = 7–8). Each point represents the mean ± SEM.

Next, we characterized the effect of two Lavandula species essential oils; *Lavandula stoechas* (*LS*), *Lavandula angustifolia Miller* (*LA*) and their active principle Linalool. Ca_V_3.2 current recordings were performed during application of increasing concentrations of *LS*, *LA* and Linalool (Fig 2). T-type current inhibition by these three compounds was concentration-dependent. Analysis of the dose-response curve after treatment with *LS* essential oil revealed IC_50_ values of 16.9 ± 2.9 μg/ml (n = 7) with a Hillslope value of 0.9 ± 0.1 (Fig 2A). TTCCs were also inhibited by serial concentrations of *LA Miller* essential oil solutions (Fig 2B). The IC_50_ value for *LA Miller* inhibition of Ca_V_3.2 currents was 34.1 ± 2.9 μg/ml (Fig 2B, n = 8) with a Hillslope factor of 1.9 ± 0.4. For Ca_V_3.2 current inhibition by Linalool, the IC_50_ value was 84 ± 8.8 μM (~12.6 μg/ml) with a Hillslope factor of 1.01 ± 0.08 (Fig 2C, n = 7).

### Effects of Lavandula essential oils and Linalool on Ca_V_3.2 channel activation

**Fig 3:**
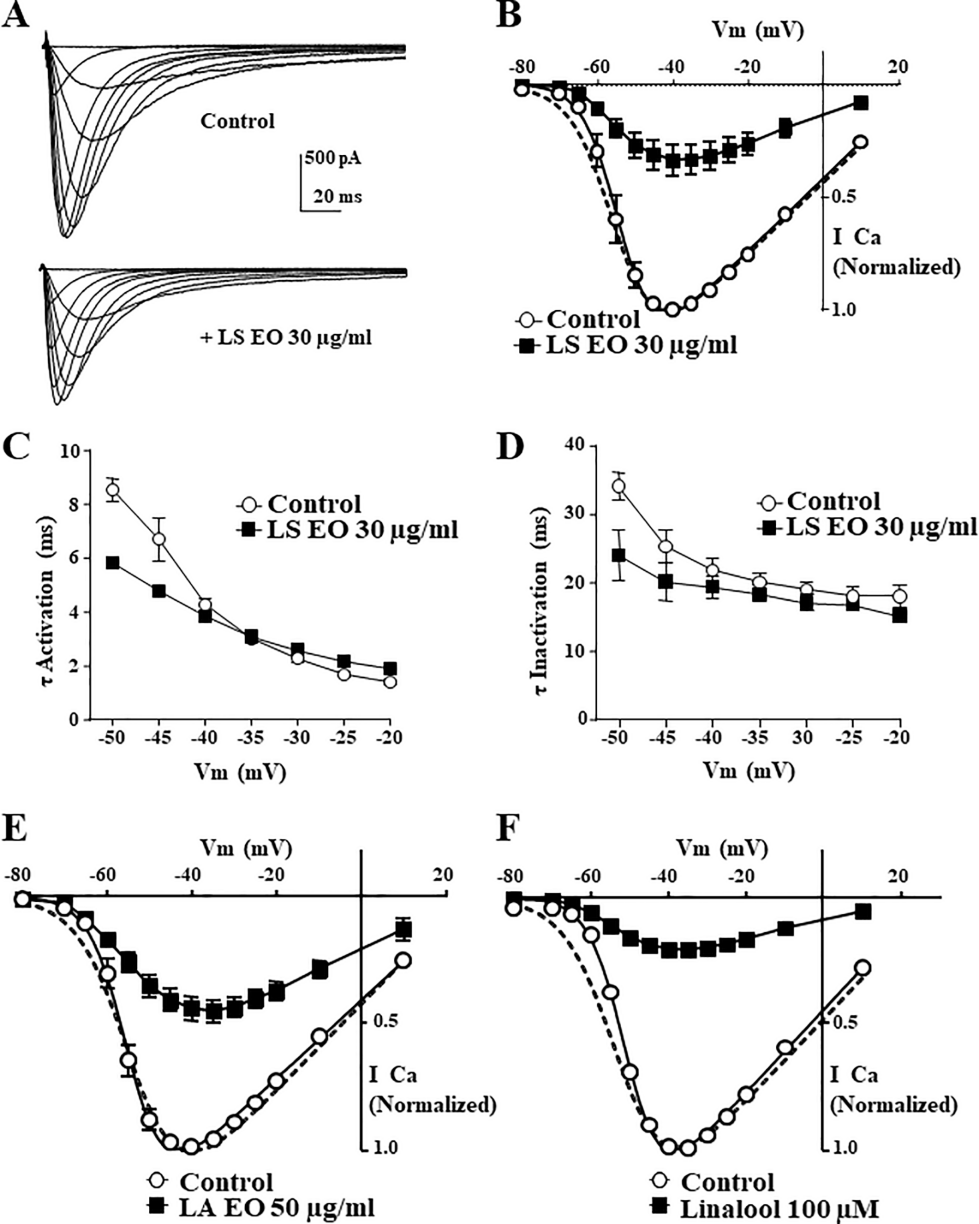
*Lavandula stoechas*, *Lavandula angustifolia* and Linalool do not affect activation of Ca_V_3.2 channels. Representative current traces of the current-voltage (*I-V*) relationship (**A**) before and after the application of 30 μg/ml *Lavandula stoechas* on Ca_V_3.2 channel (HP -80 mV). Cells were stepped to serial depolarizing 150 ms TP ranging from -80 to +50 mV (-80, -70, -65, -60, -55, 50, -45, -40, -35, -30, -25, -20, -10 and +10 mV). (**B**) *I-V* relationship of Ca_V_3.2 channels before and after 30 μg/ml *Lavandula stoechas (LS)*. Note that the *I-V* curve in the presence of *LS* is normalized to the control *I-V* curve (dotted line) for a better comparison. (**C**-**D**) Analysis of activation and inactivation kinetics (two-exponential fitting of the current traces obtained as in panel A) after treatment with *LS* showed small shifts between control and *LS*-treated recordings. (**E**) Representative *I-V* relationship of Ca_V_3.2 channels before and after 50 μg/ml *Lavandula angustifolia Miller*. (**F**) Representative *I-V* relationship of Ca_V_3.2 channels before and after 100 μM Linalool (~15 μg/ml). For a better comparison, the *I-V* relationships in the presence of the compounds are normalized (panels E and F, dotted curves). Data represents the mean ± SEM, n = 6.

Inhibition of Ca_V_3.2 channels by Lavender and its natural constituent Linalool may be related to specific modifications in Ca_V_3.2 channel gating properties. Hence, we investigated whether TTCC inhibition by Lavender could be related to change in channel availability or activation properties. Toward this goal, the inhibitory effect of these natural compounds on Ca_V_3.2 current was studied for a wide range of depolarizing test potentials (TPs) from -80 to +10 mV. Representative Ca_V_3.2 current traces before and after the application of 30 μg/ml *LS* are shown in Fig 3A (top and bottom panels respectively), as well as the corresponding current-voltage (*I-V*) curves (Fig 3B). These average *I-V* curves show that 30 μg/ml of *LS* inhibit the amplitude of Ca_V_3.2 currents similarly at all membrane potentials (Fig 3B, n = 6). Moreover, application of *LS* did not significantly shift the activation curve of Ca_V_3.2 channels. The V_0.5_ for activation was -53.8 ± 0.4 mV for control condition and -54.9 ± 0.4 mV during *LS* application, respectively, revealing no significant change in steady-state activation in the presence of *LS* (n = 6, *p* = 0.11, Fig 3B). In addition, fitting of the individual current traces, as presented in Fig 3A, revealed that neither activation nor inactivation kinetics of Ca_V_3.2 channels were changed after Lavender treatment (Fig 3C and 3D). Similar results were obtained with 50 μg/ml *LA Miller* essential oil (Fig 3E, n = 5) and 100 μM Linalool (Fig 3F, n = 6). Inhibition of Ca_V_3.2 currents following treatment with *LA Miller* essential oil and Linalool was conserved over the complete range of test potentials. Also, the threshold potential for activation and the membrane potential of the maximum peak current were the same before and during the application of *LA Miller*, with no significant shift in the activation curve of Ca_V_3.2 currents (V_0.5Ctrl_ = -54.8 ± 0.4 mV and V_0.5LA*Miller*_ *=* -54.1 ± 0.3 mV, n = 5, *p = 0*.*19*), and Linalool (V_0.5Ctrl_ = -50.5 ± 0.7 mV and V_0.5Linalool_ = -52.3 ± 0.8 mV, n = 6, *p = 0*.*14*).

### Effects of Lavandula on Ca_V_3.2 current steady-state inactivation

**Fig 4:**
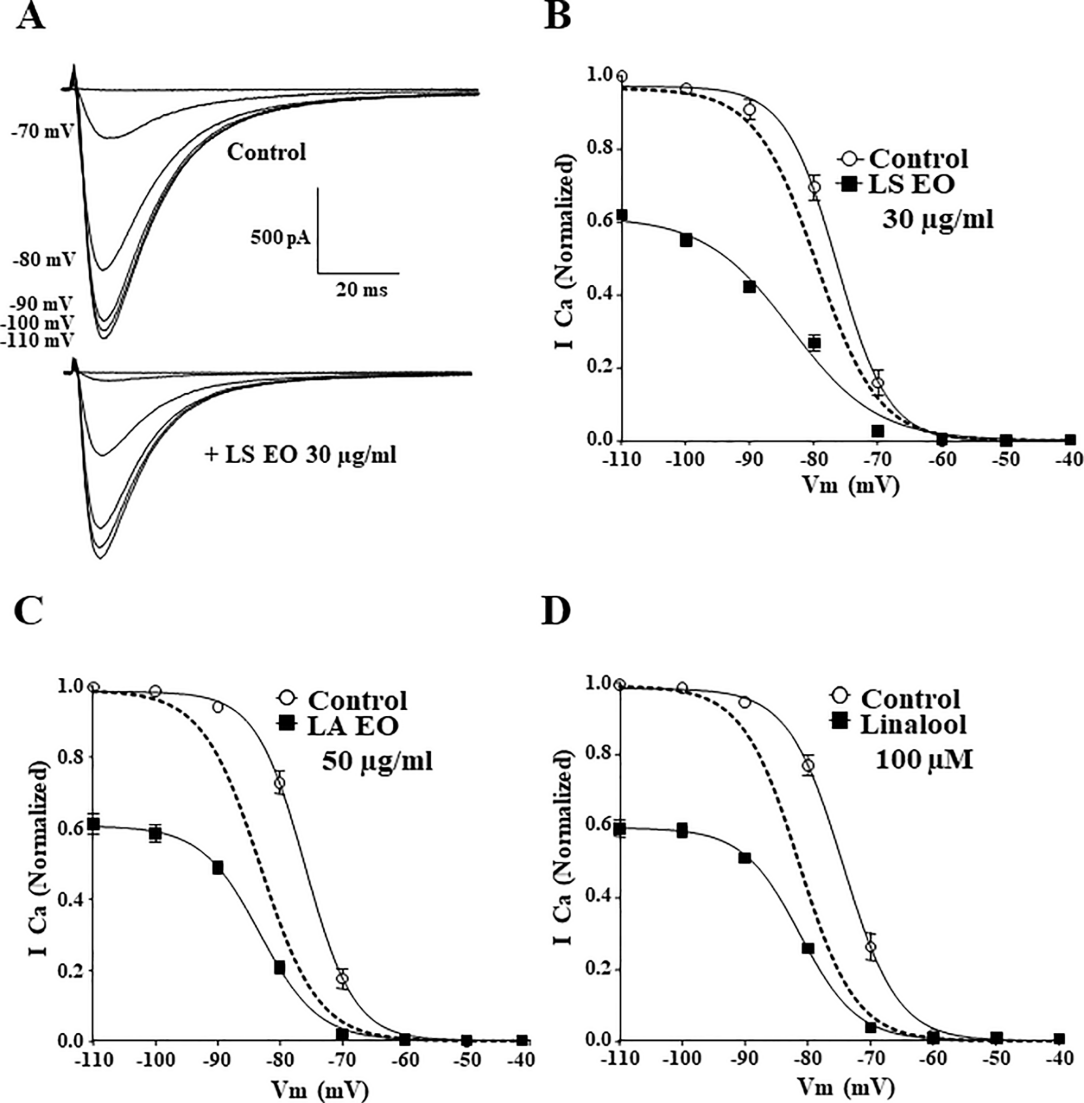
*Lavandula stoechas*, *Lavandula angustifolia Miller* and Linalool effects on steady-state inactivation of Ca_V_3.2 channels. To measure steady-state inactivation (at TP -30 mV), cells were voltage-clamped for 5 seconds at potentials between -110 and -40 mV (10 mV increments). (**A**) Representatives traces before and after treatment with 30 μg/ml *Lavandula steochas* essential oil (*LS* EO). (**B**) Steady-state inactivation before and after 30 μg/ml *Lavandula stoechas* essential oil (*LS* EO). (**C**) Steady-state inactivation before and after 50 μg/ml *Lavandula angustifolia Miller* essential oil (*LA* EO). (**D**) Steady-state inactivation before and after 100 μM Linalool (~15 μg/ml). Normalized steady-state inactivation curve in the presence of the compounds are represented by dotted curves in panels B, C and D. Data represents the mean ± SEM (n = 5–6).

**Fig 5:**
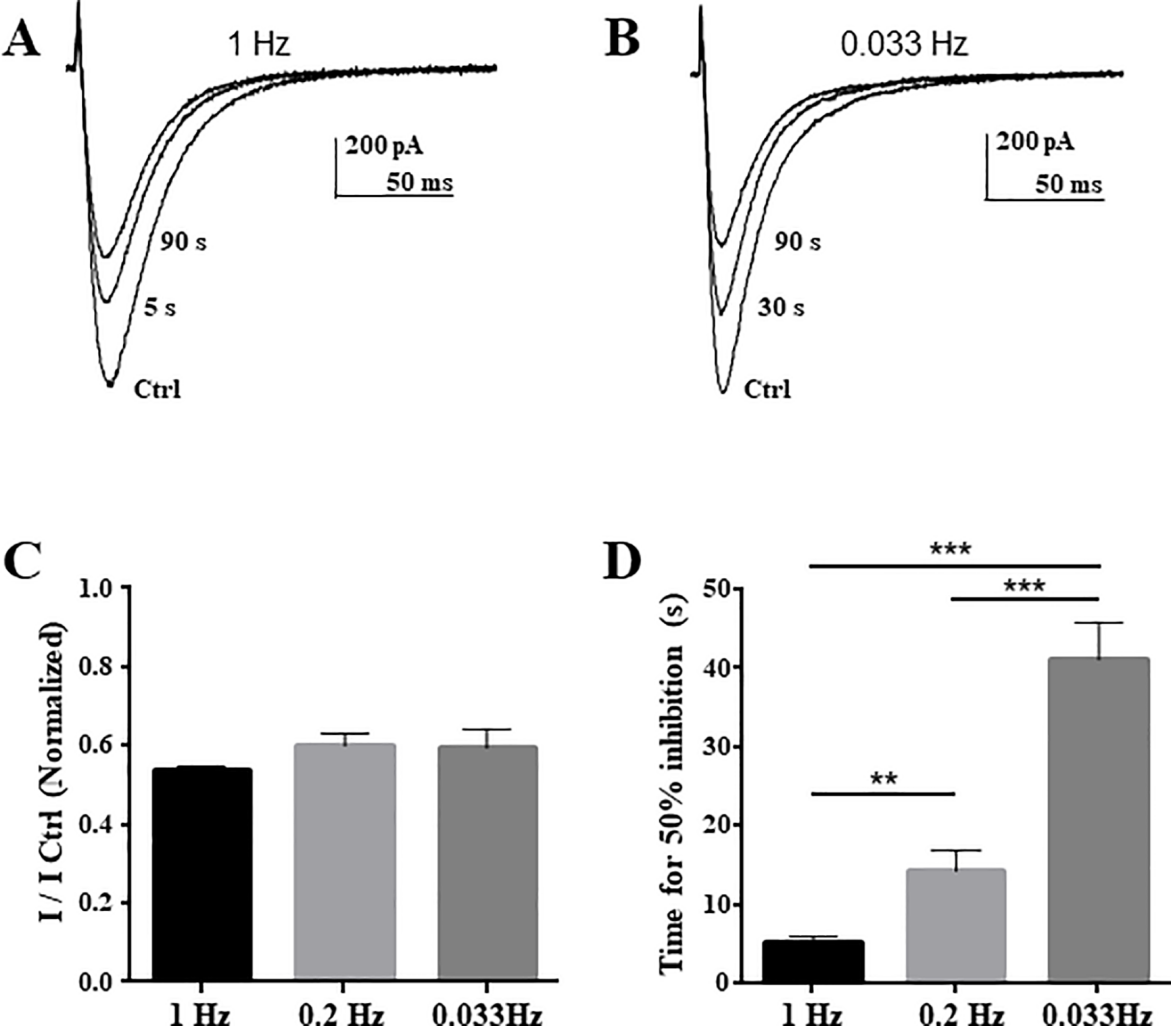
Ca_V_3.2 channel inhibition by Linalool at various frequency of stimulation. (**A-B**) Representative examples of Cav3.2 current inhibition by 100μM Linalool after 5 s (near half inhibition by linalool) and 90 s (near maximum inhibition by linalool) at 1 Hz (A) and 0.033 Hz (B). (**C**) Percentage of Linalool inhibition at three the frequencies tested, 1 Hz, 0.2 Hz and 0.033 Hz. (**D**) Time course of Linalool inhibition (Time for 50% inhibition) at the three frequencies tested, 1 Hz, 0.2 Hz and 0.033 Hz.

We next examined whether Lavender and its constituents could modify the steady-state inactivation properties of Ca_V_3.2 channels. A representative family of Ca_V_3.2 currents evoked by the protocol designed to measure steady-state inactivation is depicted in Fig 4A before (top traces) and during the application of 30 μg/ml *LS* (bottom traces). *LS* (30 μg/ml) produced a depressant action of the maximal conductance of Ca_V_3.2 channels as well as a significant hyperpolarizing shift of steady-state inactivation from -75.8 ± 1.1 mV in control conditions to -81.3 ± 1.0 mV in *LS* (n = 6, *p<0*.*05*, Fig 4B). Similar results were obtained after application of *LA Miller* (50 μg/ml, Fig 4C) and Linalool (100 μM: ~15 μg/ml, Fig 4D) with a significant shift in the steady-state inactivation from -75.9 ± 0.7 mV to -83.1 ± 0.8 mV for *LA Miller* (n = 5, *p<0*.*001*) and from -74.6 ± 0.7 mV to -81.3 ± 0.6 mV for Linalool (n = 6, *p<0*.*001*). These data demonstrate that Lavender constituents inhibit Ca_V_3.2 current by decreasing the maximal conductance of Ca_V_3.2 channels and induce a negative shift of Ca_V_3.2 steady state-inactivation curve, suggesting that Lavender compounds could interact with the inactivated state of TTCCs. In addition, we have investigated the modulation of Cav3.2 current in the presence of 100 μM Linalool at three different frequencies of stimulation (1, 0.2 and 0.033 Hz). These experiments (see Fig 5) show that, although the percentage of inhibition at the steady-state was not significantly different in all three conditions (Fig 5A–5C), the time course of inhibition was significantly faster in experiments done at 1 Hz (time for 50% inhibition ~ 5 s, Fig 5D), compared to slower frequencies: 0.2 Hz (~15 s) and 0.033 Hz (~40 s). These data, support further state-dependent inhibition of Cav3.2 channels by Linalool.

### Inhibition of Ca_V_3.2 calcium channels by Rosmarinus officinalis and Rosmarinic acid

**Fig 6:**
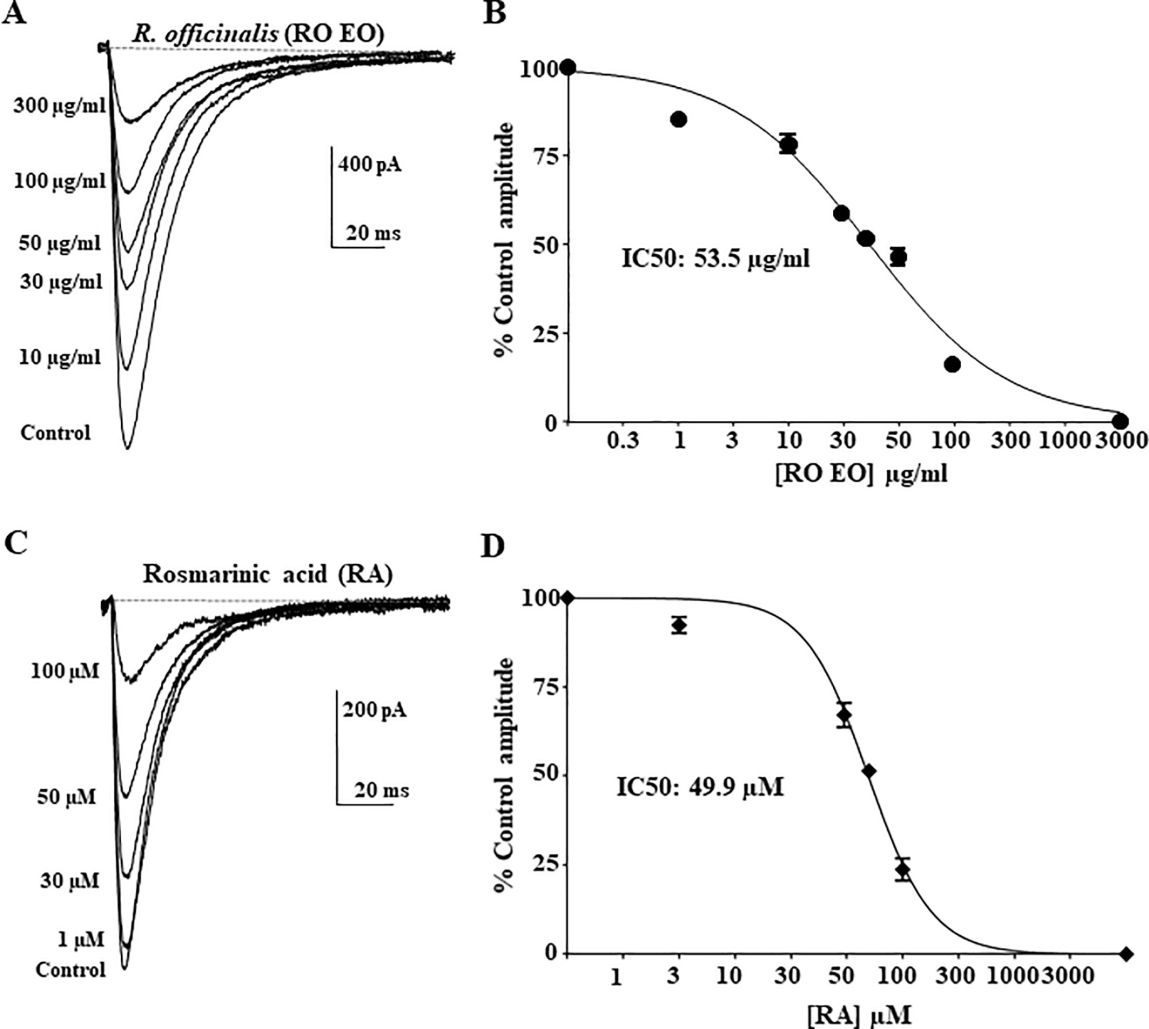
Ca_V_3.2 channel inhibition by *Rosmarinus officinalis* essential oil. Dose-response curves for *Rosmarinus officinalis* essential oil (*RO* EO) (**A-B**). and Rosmarinic acid (**C-D**). Representative current traces before and after application of serial concentrations of *RO* EO are superimposed, as shown on the left panels of each concentration-response curve. Each point represents the mean ± SEM. n = 6. The IC_50_ for Rosmarinic acid (49.9 μM) corresponds to ~18 μg/ml.

Next, we studied the effects of *Rosmarinus officinalis* (*RO*) and its active principle Rosmarinic acid (RA), a caffeic acid ester compound, on the modulation of Ca_V_3.2 channels. The Ca_V_3.2 current was inhibited by *RO* in a concentration-dependent manner (Fig 6A, n = 6). Ca_V_3.2 current inhibition by *RO* yielded an IC_50_ was of 53.5 ± 3.7 μg/ml with hillslope value of 0.7 ± 0.05 (Fig 6B, n = 6). Furthermore, RA similarly inhibited Ca_V_3.2 current in a concentration-dependent manner. The IC_50_ value was 48.2 ± 1.4 μM (~18 μg/ml) with a Hillslope value of 1.5 ± 0.2 (Fig 6C and 6D, n = 6).

### Effect of Rosmarinus officinalis and Rosmarinic acid on Ca_V_3.2 channel activation and inactivation

**Fig 7:**
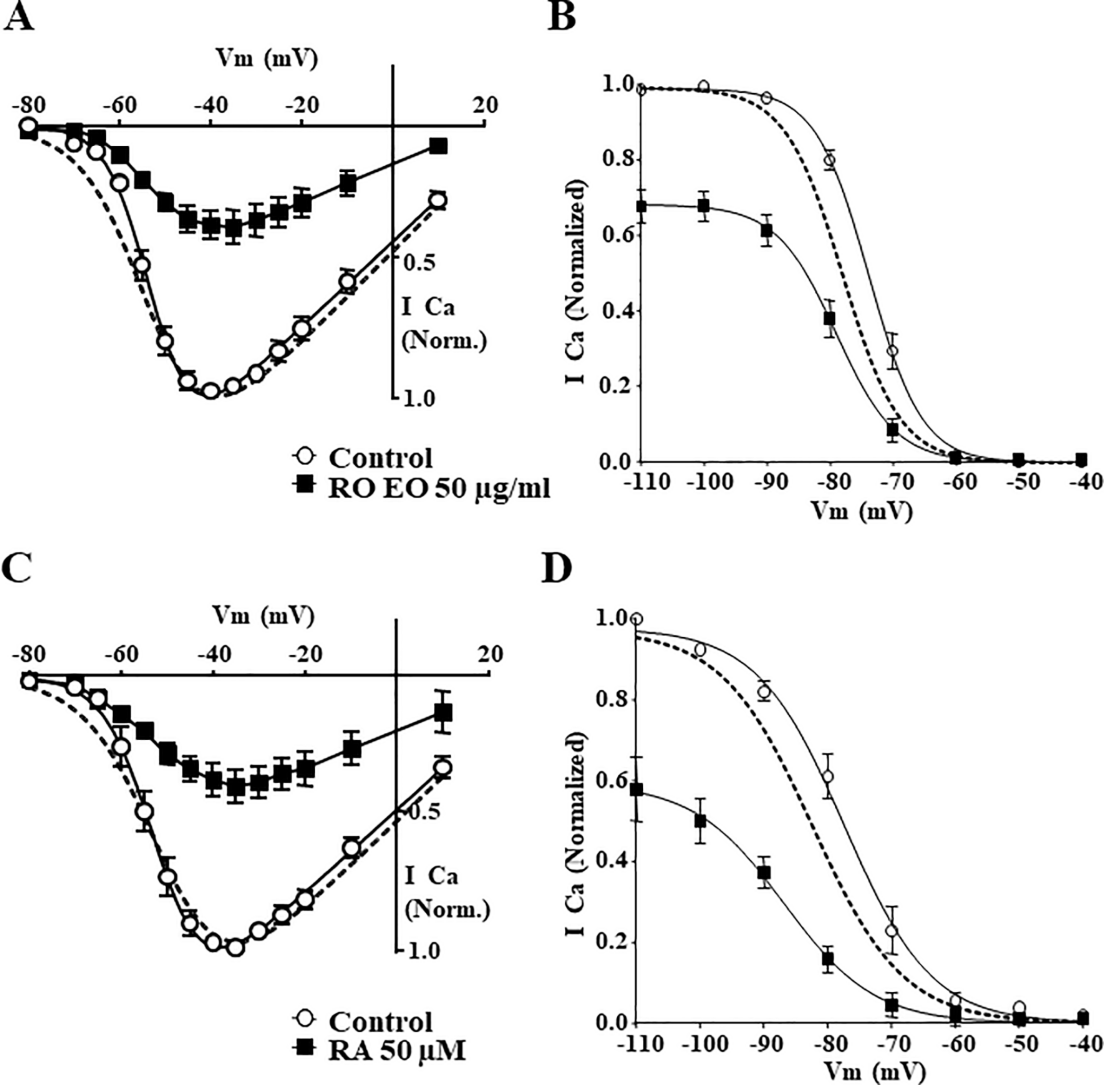
*Rosmarinus officinalis* and Rosmarinic acid affect steady-state inactivation but not activation of Ca_V_3.2 channels. (**A**) Representative *I-V* relationship of Ca_V_3.2 channels before and after treatment with 50 μg/ml *Rosmarinus officinalis*. (**B**) Representative curve of the steady-state inactivation before and after 50 μg/ml *Rosmarinus officinalis*. (**C**) Representative *I-V* relationship of Ca_V_3.2 channels before and after 50 μM (~18 μg/ml) Rosmarinic acid. (**D**) The steady-state inactivation before and after 50 μM Rosmarinic acid. Normalized *I-V* and steady state inactivation curves in the presence of *RO* and RA are represented by dotted curves in the four panels. Data represents the mean ± SEM (n = 6–7).

Similar to that described for Lavender compounds, we then studied the effect of *RO* and RA on Ca_V_3.2 activation and inactivation properties. Analysis of current traces and *I-V* curves revealed that *RO* (50 μg/ml) inhibited Ca_V_3.2 currents at all tested potentials without changing the steady-state activation properties (V_0.5Control_ = -52.8 ± 0.4 mV, V_0.5*RO*_ = -53.5 ± 0.5 mV, n = 7, *p = 0*.*3*, Fig 7A). Similar results were obtained after treatment with 50 μM RA (V_0.5Control_ = -51.8 ± 0.6 mV, V_0.5RA_ = -50.6 ± 1.3 mV, n = 6, *p = 0*.*4*, Fig 7C). To further elucidate the blocking mechanisms of *RO* and RA, steady-state inactivation was determined in the absence and presence of these natural substances. These experiments showed that treatment with 50 μg/ml *RO* both reduced the maximal conductance of Ca_V_3.2 channels and negatively shifted the midpoint of voltage-dependence of inactivation for Ca_V_3.2 towards negative potential (V_0.5Control_ = -73.7 ± 0.9 mV, V_0.5*RO*_ = -77.8 ± 1.1 mV, n = 7, *p<0*.*05*, Fig 7B). The application of 50 μM (~18 μg/ml) of RA induced a shift towards more negative membrane potentials (V_0.5Control_ = -77.4 ± 0.8 mV, V_0.5RA_ = -82.4 ± 1.2 mV, n = 6, *p<0*.*01*, Fig 7D). In addition, activation and inactivation kinetics were unchanged after either *RO* or RA treatment of Ca_V_3.2 channels.

### Lavandula steochas and Rosmarinus officinalis preferentially bind to the inactivated state of T-type calcium channels

**Fig 8:**
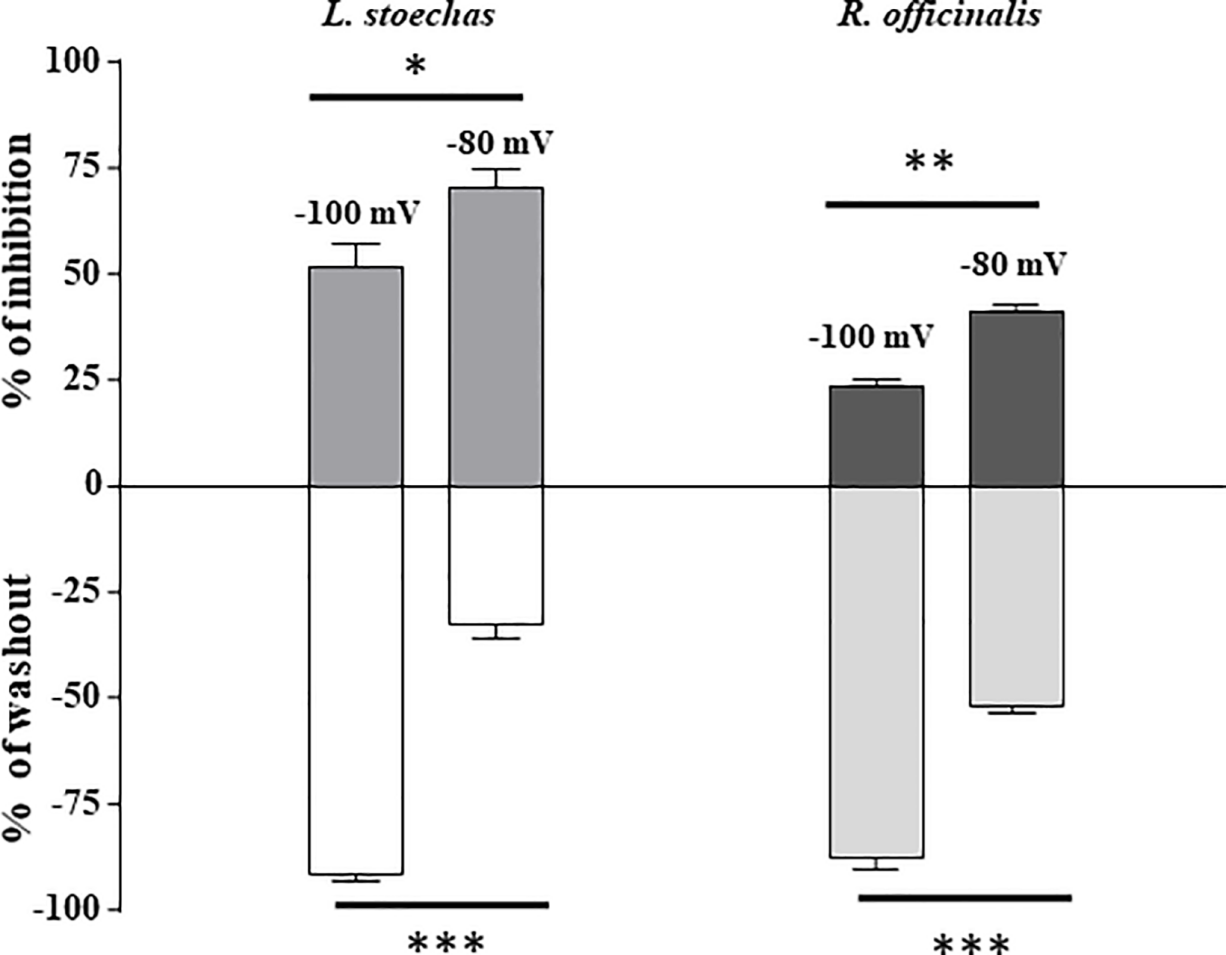
Efficacy of Ca_V_3.2 inhibition by *Lavandula steochas* and *Rosmarinus officinalis* is dependent of the resting membrane potential. Inhibition and washout of Cav3.2 channels by 20 μg/ml *Lavandula stoechas*, or 20 μg/ml *Rosmarinus officinalis* was examined for HPs of -100 mV and -80 mV. Each bar represents the average of five to six similar experiments. Data represent the mean ± SEM.

A growing body of reports suggested that TTCC blockers bind to / stabilize the inactivated state of these channels [38, 41, 42]. Indeed, the negative shift induced by Lavender and Rosemary in the channel availability suggests that these natural compounds preferentially bind to the inactivated state of Ca_V_3.2 channels, thus shifting the equilibrium away from states from which channels can open [43]. To evaluate further whether Lavender and Rosemary compounds bind to the inactivated state of TTCCs, we have measured the inhibition of Ca_V_3.2 currents by *LS* and *RS* at HPs -100 and -80 mV (Fig 8, upper graphs). If the effects of *LS* and *RO* on channel inactivation would contribute significantly to the inhibition of the Ca_V_3 channels, then applying *LS* and *RO* to cells voltage-clamped at potentials significantly more negative than -80 mV would produce less current inhibition. As expected, the inhibition by *LS* (20 μg/ml) was significantly more pronounced when cells were held at HP -80 mV (70 ± 4.4%, n = 6) than at HP -100 mV (51 ± 5.3%, n = 6, *p<0*.*05*). Similar data were obtained after the application of *RO* (20 μg/ml) with 41.3 ± 1.7% of inhibition at HP -80 mV and 23.4 ± 1.7% inhibition at HP -100 mV (n = 6 *p<0*.*01*). The efficacy of washout was examined for the two extracts at HPs -100 and -80 mV (Fig 8, lower graphs) and, conversely, washout appeared significantly more efficient at HP -100 mV (*LS*_Washout_ = 91.6 ± 1.8%, *RO*_Washout_ = 87.7 ± 3.0%) than at HP -80 mV (*LS*_Washout_ = 32.6 ± 3.1%), *RO*_Washout_ = 51.7 ± 2.5%, *p<0*.*001*). Taken together, the results suggest that these natural compounds preferentially bind to, and stabilize, Ca_V_3.2 channels in the inactivated state.

## Discussion

In this study, we describe several important findings. First, among a selection of Mediterranean medicinal plants including *Lavandula stoechas*, *Rosmarinus officinalis*, *Ricinus cumunis* and *Citrullus colocynthis*, we have identified that Lavender and Rosemary compounds could significantly inhibit the Ca_V_3.2 TTCCs in a concentration-dependent manner. Importantly, Lavender and Rosemary are widely used medicinal plants. Second, our results provide evidence that their active principles, Linalool and Rosmarinic acid respectively, also inhibit Ca_V_3.2 channels. Third, we report that these compounds induce a negative shift in the steady-state inactivation properties and we show that their inhibitory effect on Ca_V_3.2 channels is significantly enhanced in the range of physiological membrane potential (HP -80 mV), compared to more negative potential (HP -100 mV). Taken together, our findings support a pharmacological modulation of TTCCs by Lavender and Rosemary and we suggest that TTCC inhibition by these natural components may contribute to the neuroprotective and anticonvulsant activities of these medicinal plants.

Lavender (*Lavandula stoechas*, *Lavandula angustifolia Miller*) and Rosemary (*Rosmarinus officinalis*) all inhibit the amplitude of Ca_V_3.2 current in a dose-dependent manner. The IC_50_ values of *L*. *stoechas*, *L*. *angustifolia Miller* and *R*. *officinalis* were estimated to be 16.9, 34.1 and 53.5 μg/ml respectively, suggesting that Ca_V_3.2 channels are more sensitive to *Lavandula* species and in particular to *Lavandula stoechas*. Furthermore, Lavender and Rosemary also inhibit the other TTCC isoforms, Ca_V_3.1 and Ca_V_3.3. The IC_50_ values for *LA Miller* inhibition were 26.1 ± 4.8 μg/ml (n = 7) for Ca_V_3.1 and 86.2 ± 18.1 μg/ml for Ca_V_3.3 (n = 7). Interestingly, the percentage of inhibition obtained after treatment of the various TTCCs with 10 μg/ml of *LA Miller* (31% for Ca_V_3.1, 16% for Ca_V_3.2 an 10% for Ca_V_3.3) is similar to that obtained after treatment of HVA, P/Q-type calcium channels with Silexan (25%), a patented active substance produced from *L*. *angustifolia* flowers by steam distillation and consisting of the main active constituents linalool and linalyl acetate [10]. Altogether, our data extend previous electrophysiological studies describing the effect of Lavender and its active principle Linalool on other voltage-gated calcium channels [10, 44]. Importantly, we report for the first time to our knowledge, inhibition of voltage-gated calcium channels, in particular TTCCs, by *Rosmarinus officinalis* and its active principle Rosmarinic acid.

Linalool is a monoterpene compound reported to be the major component of Lavender essential oil. It has been reported to trigger glutamate activation in response to NMDA receptors modulation in the cerebral cortex [45] and reduces acetylcholine release at mouse neuromuscular junction by modifying nicotinic receptors kinetics [46], suggesting possible pathways in sedative and anticonvulsant effects in mice [47, 48]. Other studies to investigate the molecular mechanisms associated with linalool therapeutic use revealed that Linalool could interact with voltage-gated channels, in particular voltage-gated calcium channels [10, 44]. Narusuye et al. found that Linalool non-selectively suppressed the voltage-gated currents I_Ca,L_, I_K_, I_A_, and I_Ka_ in retinal horizontal cells as well as the currents I_Na_, I_Ca,L_, I_K_, I_A_, and I_K(Ca)_ in retinal ganglion cells. Fura-2-based calcium imaging technique was used to test the effect of linalool on newt olfactory receptor cells (ORC) expressing both I_Ca,L_ and I_Ca,T_ [44] and showed that 3 mM Linalool reversibly inhibited calcium currents in ORC by 44.9 ± 2.6%. Similarly, Schuwald et al. reported a decrease on KCl-induced calcium influx in murine synaptosomes after treatment with linalool and linalyl acetate concentrations (1 μM), suggesting potent anxiolytic properties of linalool via modulation of voltage-dependent calcium channels [10]. Our electrophysiological study confirms the inhibition of voltage-gated calcium channels, specifically TTCCs, by Linalool. Linalool attenuates Ca_V_3.2 currents in a dose-dependent manner. The IC_50_ for Linalool inhibition of TTCCs estimated to be 84 μM is found to be lower than the IC_50_ obtained for the inhibition of other ionic channels in different preparations. As an example, the IC_50_ values of Linalool blockade for the voltage-gated sodium is estimated to be around 560 μM [49], suggesting that Linalool is more potent in inhibiting Ca_V_3.2 channels.

Rosmarinic acid has been shown to exert neuroprotective effect against antioxidative stress and excitotoxicity and to possess anxiolytic/antidepressive-like effects [20, 50]. The mechanism by which RA exerts its anti-inflammatory effects is not well understood, although it has been shown that RA inhibits lipoxygenase [51] and cyclooxygenase activity [52], block complement activation [53] and T-cell antigen receptor (TCR)-mediated signaling [54]. Whether Rosmarinic acid could modulate ion channels, especially voltage-gated channels, was currently unknown. Our study therefore reveals that TTCCs, are inhibited by *Rosmarinus officinalis* and Rosmarinic acid in a dose- and voltage-dependent fashion. Consequently, TTCCs may therefore represent a novel molecular target for Rosmarinic acid, although further experiments are needed to characterize the efficacy of Rosmarinic acid to possibly modulate other ion channels.

Inhibition of TTCCs is highly dependent on their inactivation state. Analysis of the biophysical properties of Ca_V_3.2 channels before and after Lavender (*Lavandula stoechas*, *Lavandula angustifolia Miller* and *Linalool*) and Rosemary *(Rosmarinus officinalis* and Rosmarinic acid) treatments showed that these natural compounds not only reduced the maximal conductance of Ca_V_3.2 channels but also shifted the steady-state inactivation properties towards more negative membrane potentials without having effect on the activation properties. Our study describes that inhibition of Ca_V_3.2 channels by Lavender and Rosemary was significantly enhanced for HP -80 mV, compared to HP -100 mV (Fig 7). Indeed, the blocking effect was more efficient at a HP mimicking resting’s membrane potential, at which a large fraction of TTCCs are inactivated [42, 55]. This suggests an interesting mechanism by which Lavender and Rosemary could attenuate the cell excitability by decreasing intracellular calcium concentration and inducing sedative and/or anticonvulsant-like effects, as well as other various therapeutic effects such as neuroprotective properties. Our results showing that these compounds negatively shift the inactivation state suggest that these natural compounds interact with inactivated TTCCs and stabilize them in the inactivated state. This is reminiscent to that reported for phenylalkylamines and dihydropyridines that bind preferentially to the inactivated state of L-type calcium channels (HVA), conferring tissue-selectivity of these drugs that are useful as antihypertensive and antiarrhythmics treatments [56, 57].

Compounds selective on TTCCs could have unexpected therapeutical utility, particularly to treat the various disease states in which TTCCs are up‐regulated. For instance, up-regulation of Ca_V_3.2 channels was observed in both cardiac myocytes and chromaffin cells maintained under chronic hypoxic conditions [58, 59]. Cav3.2 channel overexpression was also found associated to neuroendocrine differentiation of prostate cancer cells [60]. Importantly, TTCCs represent novel interesting molecular targets for pain and epilepsy [31, 33, 43, 61–63]. Inhibition of TTCCs has been reported to play an important role in the therapeutic action of many drugs [64]. For example, Gomora et al. confirmed the hypothesis that the blockade of TTCCs may underlie the therapeutic usefulness of succinimide antiepileptics [65]. In the same context, Tringham and coworkers have identified two high affinity TTCC blockers that were able to attenuate burst firing of thalamic reticular nucleus neurons in the Genetic Absence Epilepsy Rats from Strasbourg (GAERS) [66]. Blockade of TTCCs is suggested to be useful in a wide variety of neurological disorders such as neuropathic pain [67, 68]. Indeed, Jagodic and collaborators have demonstrated that TTCCs are significantly upregulated in small dorsal root ganglion (DRG) during chronic constriction injury (CCI)-induced neuropathy [69]. Therefore, the inhibition of TTCCs by NNC 55–0396, a selective TTCC inhibitor [70] was suggested to be useful in decreasing pre- and postsynaptic transmission and the neuronal activity in anterior cingulate cortex after a CCI leading to the attenuation of neuropathic pain [71]. ABT-639 is a peripherally acting TTCC blocker that selectively inhibits TTCCs in a dose-dependent manner. In preclinical studies, oral administration of ABT-639 was reported to alleviate nociceptive and neuropathic pain in rat models [72]. However, phase 2 clinical studies using microneurography, a relevant technique that assesses abnormal spontaneous activity in C-nociceptors as a marker for spontaneous pain, revealed that administration of ABT-639 100 mg twice daily did not reduce neuropathic pain in diabetic patients [73, 74]. Interestingly, Z944, a potent selective blocker of TTCCs (50–160 nM) was shown to be effective in reducing pain in preclinical models as well as in human patients [75].

Other studies have also suggested TTCCs as interesting molecular targets for natural compounds. Eugenol, a local analgesic used in clinical dentistry that naturally present in cloves (*Syzygium aromaticum*) modulates TTCCs in a dose-dependent fashion with IC_50_ of 500 μM. The depressant effect of Eugenol on TTCCs was suggested to inhibit action potentials and the neuronal conduction of sensory signals in TG neurons leading to eugenol pain-relieving action [38]. Furthermore, Ross et al. have demonstrated that Δ9-tetrahydrocannabinol and cannabidiol, the most prevalent biologically active constituents of *Cannabis sativa*, inhibit recombinant as well as native TTCCs [39]. Interestingly, Cannabidiol is currently under development as an antiepileptic drug [76]. It is likely that attenuation of TTCC conductance causes the decrease in neurotransmitter release mediated by these compounds contributing to the well-known psychoactive actions of cannabinoids, as well as the anti-nociceptive and anticonvulsant properties [39, 76–78].

## Conclusion

Our data show that Lavender and Rosemary extracts efficiently inhibit TTCCs by preferentially binding to inactivated channels. Altogether, this study demonstrates that TTCCs represent a novel molecular target for Lavender and Rosemary likely to be involved in some of the Mediterranean medicinal plants’ therapeutic use.

## Abbreviations

TTCC: T-type calcium channel
Cav: voltage-gated calcium channel
HEK: human embryonic kidney
GFP: green fluorescent protein
HP: holding potential
